# Vertical Root Fracture: Preservation of the Alveolar Ridge Using Immediate Implants

**DOI:** 10.1155/2014/520169

**Published:** 2014-03-12

**Authors:** Edmar de Oliveira Oya, Debora Pallos, Humberto Osvaldo Schwartz-Filho, William Cunha Brandt, Wilson Roberto Sendyk, Caio Vinicius Gonçalves Roman-Torres

**Affiliations:** Department of Implantology, University of Santo Amaro, Rua Prof. Eneas Siqueira Campos 340, 04829-300 São Paulo, SP, Brazil

## Abstract

Teeth with vertical root fracture (VRF) have complete or incomplete fractures that begin in the root and extend toward the occlusal surface. The most frequent causes of VRF originate from physical trauma, occlusal prematurity, inadequate endodontic treatment, and iatrogenic causes. Diagnose is difficult and delay can cause stomatognathic system problem. The purpose of this case report was to evaluate immediate implant placement after extraction of teeth with vertical root fracture. For the 1st case, the VRF in 1st left lower molar was confirmed during surgical flap and at the same time, the tooth was removed and immediate implant was placed. For the 2nd case, the VRF 1st left lower molar was confirmed during endodontic access and at the same appointment, the tooth was removed and the immediate implant is placed. Several studies have shown that immediate implants have similar success rates when compared with late implants. Consider that this approach is a safe procedure with favorable prognosis. In cases of VRF, the main factor to be considered is the presence of adequate bone support and immediate implants can preserve the vertical bone height, adding the fact that good patient compliance reduces the number of surgical interventions and promotes the functionality of stomatognathic system.

## 1. Introduction

Vertical root fracture (VRF) according to the American Academy of Endodontics is only located in the root portion dental, directed buccoingual/palatal and is treating the removal of the dental element placement of a fixed or removable prosthesis or a placement implant osseointegrated [1].

A vertical root fracture can present the complete or incomplete form, extending the root portion which may protrude into the enamel to the long axis of the dental element. Usually it extends from the pulp to the periodontal ligament, affecting more often the proximal surfaces. In most cases, a definitive diagnosis of VRF can only be done by periodontal probing, radiographic, and surgical exposure (inspection of the root surface). The most frequent causes of VRF originate from physical trauma, occlusal prematurity, poor endodontic treatment, and dental treatment iatrogenic. The patient often does not have the classic symptoms, masking the diagnosis, which can aggravate the treatment. Maintaining the adjacent bone tissue is important and local bone loss may be related to the time when the patient presents the fracture and local contamination will promote an inflammatory process in the region followed by bone loss. In cases of VRF, the main factor to be considered is the presence of adequate bone support for determining the prognosis. But when we find conditions favorable, alveolar bone affected plus the ability to restore the system stomatognathic immediate implant placement may be the therapy of choice.

Some authors evaluated the vertical root fractures in teeth without endodontic treatment. The results showed that fractures occur frequently in the first molars and premolars of individuals of 40–69 years, the prevalence was two times higher in men than in women, caused by excessive and repetitive chewing force [2, 3]. Teeth without endodontic treatment, the VRF begins at the apex and occurs in buccolingual direction with minimal discomfort. Over time, normal chewing painful symptoms should appear, causing the separation of the fragments [4].

Root fractures in endodontically treated teeth may exhibit lateral radiolucency, small fistula localized periodontal pocket, and gingival tissue in the tooth evaluated and these are signs that may aid in the correct diagnosis [5].

The presence of vertical fractures has a poor prognosis and leads to extraction of the affected tooth. The factors related to VRF are bone loss, pain on percussion, extensive restorations, and a predilection for the women and older individuals [6].

Some factors such as teeth with incomplete apex and root canal treatment, removal of tooth structure, endodontic sealing inappropriate, and unbalanced occlusion can lead to VRF. An accurate diagnosis must be based on information about the patient's medical history, dental history, and also signs and symptoms and radiographic diagnosis made by the clinician [7]. Occasionally signs and symptoms and radiographic findings may promote confusion and be interpreted wrongly [8]. There is a difficulty in diagnosing the fracture and delay can lead to bone loss, pain, and problems in the stomatognathic system [9].

The diagnostic VFR concluded that it is necessary to incorporate additional tests to the clinical and radiographic diagnosis. Some studies evaluated the efficacy of cone-beam computed tomography and periapical radiographs in detecting vertical fractures in endodontically treated teeth extracted recently. The results showed greater accuracy for CT than for periapical radiographs in detecting vertical fractures and the presence of sealer material does not diminish the accuracy of CT in the detection of vertical fractures [8–11].

Root fractures generally have the apical direction, and most of the cracks (stage before the fracture) are present immediately after endodontic treatment, starting in the inner portion of the canal heading for the outer portion, spreading to the coronal portion [12].

The pattern of alveolar bone loss associated with VRF in endodontically treated teeth was evaluated [13]. Bone defects always accompanied the fracture line and in cases of dubious or inconclusive diagnosis, an exploratory surgery is indicated.

Some studies have evaluated the clinical results in immediate implants in teeth with signs of RVF. They concluded that the use of immediate implants can be considered as safe and effective after extraction by VFR [14–20]. However one of the contraindications for immediate implants is the presence of infection in the region of the tooth to be extracted with FVR [21, 22].

One of the principal parameters to be observed in the immediate placement of implants in the case of VRF is the remaining bone architecture. The level of bone crest and the inner wall of the socket should be accessed through direct internal probing and viewing. The decision of the immediate installation of the implant should be taken right now. The placement of implants will depend on the location and extent of root fracture with consequent bone loss.

The aim of this study was to evaluate the possibility of immediate placement of implants in dental alveolar with RVF through the report of two clinical cases.

## 2. Case Description

The study was designed as a single-center prospective clinical case series in which patients with a vertical root fracture were included, treated, and followed up. The study was approved by the ethical committee of the University of Santo Amaro, SP, Brazil, under reference 295.916. At the first visit, all patients were properly informed of the nature of the study and a written informed consent was obtained.

### 2.1. Case 1

**Figure 1:**
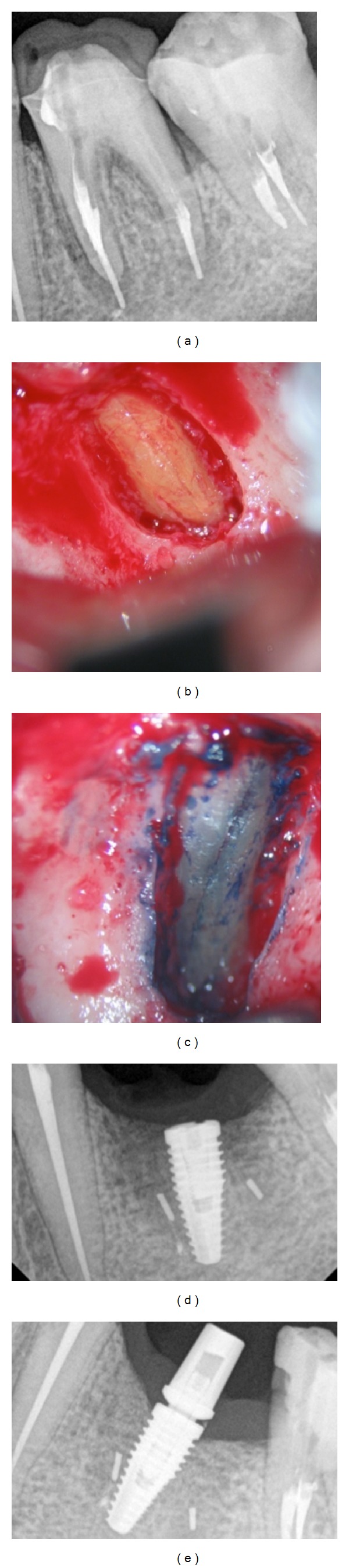
Initial radiograph of the first molar. Observe the extravasation of the filling material in the mesial root (a). Clinical aspect of the mesial root showing the vertical root fracture and bone fenestration (b). Application of dye (SableSeek, Ultradent) confirming the presence of RVF (c). Radiograph aspect after immediate implant placement (Neodent, 10.0 × 4.3) (d). Radiograph aspect 4 months after the immediate implant installation with the placement of temporary crown (e).

A 42-year-old male patient, nonsmoking, without any history of systemic disease was referred for the private dental office for endodontic surgery in the 1st left lower molar. He had no pain symptoms and the clinical characteristics after the periodontal probing evaluation showed that he is healthy, but with the presence of fistula in buccal region. The periapical radiographs (Figure 1(a)) indicated overfilling. A full-thickness gingival flap preserving the gingival margin for endodontic access was performed after initial curettage was possible to visualize the vertical root fracture (Figure 1(b)). Through a dye Sable Seek (Ultradent, South Jordan, Utah 84095, USA) was confirmed fracture (Figure 1(c)). At the same time of surgery, the extraction and immediate postextraction implant placement was performed (Figure 1(d)). After preparation of the site, an implant of 4.3 mm in diameter by 10 mm long was installed (Neodent, Curitiba, PR, Brazil).

After surgery, patient had mild swelling and no discomfort during the healing period. On the day of surgery, the patient received 2 g of amoxicillin 1 hour before surgery [23]. After placement of the implant, a marginal defect area surrounding the implant was filled with grafted deproteinized bovine bone Bio-Oss (Geistlich AG, Wolhusen, Switzerland). After a healing period of about 4 months, a screw-type implant-supported provisional restoration was placed, and the implant started occlusal loading (Figure 1(e)).

### 2.2. Case 2

**Figure 2:**
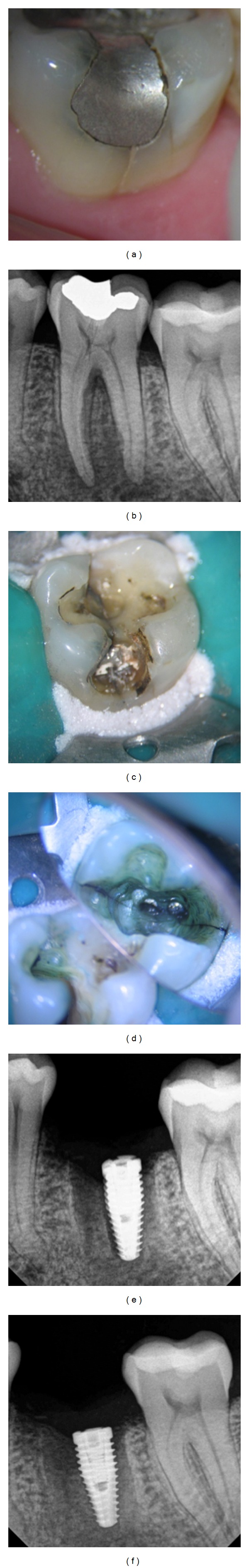
Initial clinical aspect. Note the fracture in vestibular region below the end of restoration (a). Radiograph aspect with diffuse radiolucent image (b). Clinical aspect after removal of restoration (c). Application of dye (SableSeek, Ultradent) confirming the presence of RVF (d). Radiographic image after installation of the immediate implant (Neodent, 10.0 × 4.3) (e). Radiograph aspect 4 months after immediate implant installation (f).

A 39-year-old female patient, nonsmoking, without any history of systemic disease, was referred by the surgeon to clinical endodontic treatment of the 1st left lower molar. During the clinical examination with the aid of the microscope (DF Vasconcellos, São Paulo, SP, Brazil), was shown a crack at the crown on the labial surface without the presence of periodontal pockets (Figure 2(a)). Vitality tests on tooth (thermal hot and cold) were negative and vertical percussion pain was present. Radiographic examination showed a diffuse, radiolucent periapical image. (Figure 2(b)). The restoration was removed enabling the visualization of the crack toward the pulp chamber (Figure 2(c)). When opening the pulp chamber, the emptying with curettes and irrigation with sodium hypochlorite was performed. With use of dye SableSeek, it was possible to visualize the fracture in buccal-lingual direction (Figure 2(d)), indicating the tooth for extraction with immediate implant installation performed in the same time (Figure 2(e)). An atraumatic extraction was performed without flap elevation to preserve the integrity of the remaining buccal and lingual bone plates. After preparation of the site, an implant of 4.3 mm in diameter by 10 mm long was installed (Neodent, Curitiba, PR, Brazil). After placement of the implant, a marginal defect area surrounding the implant was filled with grafted deproteinized bovine bone Bio-Oss (Geistlich AG, Wolhusen, Switzerland). The patient had mild swelling and no discomfort during the healing period. After a healing period of about 4 months, a screw-type implant-supported provisional restoration was placed, and the implant started occlusal loading (Figure 2(f)).

## 3. Discussion

In general, implant is considered only after complete healing of the extraction wound, and proper healing period is required after implant placement; therefore, the overall treatment period is long. Recently, implant placement in fresh extraction sockets has been reported, and clinical guidelines involving immediate implant placement have been proposed to give patients options to achieve the ideal outcome.

In this study, we can observe the successful placement of implants in the two cases presented. In Case 1, the VRF was confirmed after the gingival flap surgery, evidenced by the dye and visualized with the aid of the operating microscope. In Case 2, the VRF was confirmed during access for endodontic treatment, evidenced by the dye. In both cases, the subjects had pain symptoms, positive and negative vertical percussion for the remaining vitality tests. In Case 1, it was possible to observe changes in the adjacent periodontal tissues, probably because the time of occurrence VRF in this case was 3 months (as reported by patients). In Case 2, the individual was indicated for endodontic treatment because of pain, and during the procedure of surgical endodontic access VRF was diagnosed, without compromising the periodontal tissues and the time between the onset of painful symptoms and diagnosis was 7 days as the cases presented to VRF may occur both in endodontically treated teeth (Case 1) as in teeth without treatment (Case 2).

A vertical root fracture manifests as a line of complete or incomplete fracture extending obliquely or longitudinally along the tooth root portion [7, 10, 12]. Vertical root fractures usually result in extraction of the affected tooth making it a complex problem resolution in daily clinical practice, as often happens unexpectedly, causing aesthetic problems and chewing [13, 24].

The VRFs are often associated with premolars and mesial roots of molars. Several authors reported rates greater than 64% in the prevalence of these teeth to FRVs, that is, having roots in flattening mesiodistally, consequently a lower thickness in buccolingually. So the fracture line is initially located on the buccal or palatine teeth affected [2, 5, 25].

VRF could occur due to some factors such as occlusal imbalance, cross bite, eating habits, and excessive wear during endodontics treatment [4, 5, 12]. However removal of dentin endodontics practically was not necessarily related to increased susceptibility to VRF [6]. The most frequent causes of FRVs related to endodontics are performed the pressure with finger spreader during lateral condensation endodontic fillings [3, 9, 24, 26] and excessive wear during endodontic treatment [3]. Due endodontic therapy is the inevitable removal of dentin for root canals making access to both the coronal portion as root susceptible to fractures. In teeth with pulp vitality, the strength of occlusion will be decisive in cases of VRF.

It is very difficult to identify clinically the presence of VRF, especially in the early stages. The most common signs described in the literature are the presence of localized periodontal pockets [2]. Fistulas combined with deep pockets mainly by vestibular described by some authors showed that incidence of 35% and 42%, respectively [5, 27]. In the case of VRFs no pathognomonic sign, but a series of signs, symptoms and radiographic features that make quick and decisive diagnosis.

The protocol for the placement of intraosseous implants is recommended a standby time of up to 6 months after extraction [21]. Thus, there may be a reduction in bone volume and height which helps to reduce the possibility of the installation of dental implants after this timeout [20]. Some authors do not indicate the placement of immediate implants when there is presence of infection in the socket to be implanted, as there is the potential for contamination during bone integration due to infection present within the process [22].

Moreover, several studies have demonstrated that immediate implants have the same levels of success late implants. Immediate implants can preserve the vertical bone height, adding the fact that good patient compliance reduces the number of surgical interventions and promotes all the functionality stomatognathic systems [14–20].

The importance of debridement in cases of infected wells was reported by some authors and the total removal of tissue inflammation/infection in the alveoli before inserting the implants is vital and had almost 100% success. The immediate implant placement depends on an excellent debridement for the elimination of any contamination in the tissues [15–17].

In the presence of periapical pathology, a decision must be made quickly, or the strategy of immediate implant placement must be aborted. The diameter of the periapical lesion must be taken into account as lesions with diameter larger than the implant placement more apically requiring three to four millimeters are sufficient to stabilize the implant [16]. There is great difficulty in the classification of bone defects, classifications currently used do not include cases of RVF or when they detect the RVF, is not involved the placement of dental implants [28, 29].

The diagnosis of RVF can be problematic and should contain as much information as possible to facilitate the prognosis and treatment plan. It is usually associated with signs and symptoms that the patient may report and the dentist has to have the knowledge to properly diagnose. Once removed, the fractured tooth dental surgeon must evaluate the architecture end of the remaining bone and thus determine whether it is possible to place the implant immediately or propose a regeneration therapy for other surgical procedures. If there is an inappropriate bone support, installing the implant must be extended and the use of regenerative bone grafting procedures and/or use of membranes are recommended in the treatment of alveolar to maintain the dimensions of the alveolar ridge, which will facilitate the future installation of the implant, providing a more favorable prognosis in relation to the final outcome of the prosthesis on implant.

## 4. Conclusion

The immediate implant placement depends on the extent of bone destruction, and there are parameters in the literature to guide the clinician to take the appropriate decision quickly, since in most cases, the destruction is only confirmed during exploratory surgery.

## 5. Clinical Significance

In cases of VRF, the main factor to be considered is the presence of adequate bone support and immediate implants can preserve the vertical bone height, adding the fact that good patient compliance reduces the number of surgical interventions and promotes the functionality of stomatognathic system.
